# Foreword

**Published:** 2013-12

**Authors:** Carissa F. Etienne

**Affiliations:** Director Pan American Health Organization

Food safety is a global priority and an important public-health issue as foodborne diseases (FBDs), infections, and intoxications are a major cause of morbidity, mortality, and economic burden worldwide. Recognizing the importance of food safety in preventing and controlling FBD as an important cause of illness and deaths worldwide, the World Health Organization (WHO) adopted Resolution WHA 53.15 in 2002, establishing food safety and the prevention and control of FBD as a global priority. Further, in May 2010, the WHO adopted Resolution WHA 63.3 urging its Member States to improve evidence-based food safety efforts by conducting disease burden studies. Similarly, the Pan American Health Organization (PAHO), as the WHO Regional Office for the Americas, included food safety as one of its strategic objectives in its 2008-2012 Strategic Plan and FBD Surveillance as one of the five components of its hemispheric Plan of Action for Food Protection.

Precise information on the burden of illness (BOI) is needed to help guide the FBD control efforts. In response to the gap in data, the WHO launched an initiative to estimate the global burden of FBD in collaboration with multiple partners in 2006 and developed a rigorous approach for BOI estimation worldwide. For several years, PAHO/WHO has been catalyzing the creation of capacity in Member States to quantify the national burden of foodborne diseases. Recently, some countries in the Americas published estimates of foodborne disease burden. Nevertheless, in large parts of the Americas, such estimates are completely lacking.

In the Caribbean countries, the epidemiology of food- and waterborne illnesses at the community level is poorly understood. Little information on disease burden and aetiology is available, thereby limiting appropriate prevention measures. To better understand the epidemiology of FBD, measure its burden and impact, and thereby develop appropriate prevention and control measures, PAHO/WHO and the Caribbean Epidemiology Centre (CAREC), in collaboration with Member States and strategic partners, executed a Caribbean BOI study from 2008 to 2012 in seven countries (Barbados, Dominica, Grenada, Guyana, Jamaica, Saint Lucia, and Trinidad and Tobago). These BOI studies provide evidence-based information to guide the allocation of limited resources intended for the health agenda, appropriate intervention measures, and to ensure the sustainability of the Caribbean tourism economies.

At the time when these studies were done, CAREC was a PAHO/WHO centre. As of January 2013, CAREC became part of the Caribbean Public Health Agency (CARPHA), the latter being governed by the Caribbean Community (CARICOM). CARPHA continues with the responsibility for preventing disease, promoting heath, and responding to public-health priorities in the Caribbean region. The strategic partners in the Caribbean BOI studies were: Public Health Agency of Canada, University of Laval in Canada, University of the West Indies in Trinidad, Ross University in Dominica, St. George's University in Grenada, and the Caribbean Eco-Health Programme (CEHP). The CEHP was designed to address the challenge of building capacity and capabilities, translating and transferring knowledge, technology, and skills to public and environmental health professionals working in the CARICOM region. The PAHO/WHO and CAREC-led Caribbean BOI studies were one of four components of the CEHP. Funding was provided by PAHO, CAREC, Member States, and largely by a grant from the International Development Research Centre, Ottawa, Canada, that was obtained by the CEHP.

As of June 2012, the BOI studies estimated the prevalence and burden of acute gastroenteritis (AGE), specific FBD pathogens, risk factors of infections, economic costs, and gaps in AGE surveillance and, thus, provided information to guide appropriate FBD reduction efforts and food safety policy interventions for the seven countries in the Caribbean region. This Supplement of JHPN presents the findings of the burden of illness studies in respective countries. All countries used a similar methodology to conduct the population-based and laboratory-based surveys. Using a series of multipliers derived from the two national surveys, the burden of AGE, FBD, and specific FBD pathogens was calculated. Saint Lucia conducted the first BOI study. Lessons learnt from this pilot study were used in improving the implementation of the studies in other countries of the region.

These BOI studies are the first to be conducted by the Caribbean Member States to estimate the prevalence and burden of AGE and FBD. The findings are profound and have provided valuable information to guide appropriate FBD reduction efforts and food safety policy interventions for the Caribbean. This syndromic approach is a relatively simple and low-cost initial step in gathering reliable information on the burden of disease associated with food consumption. However, we recognize that complementary approaches are needed to define better the burden of diarrhoeal illness associated specifically with the transmission of foodborne pathogens. Foodborne diseases are an important cause of morbidity and mortality in the Caribbean. The calculation of the cost of unsafe food, and especially of the burden arising from microbiological contaminants in food, is a starting point for determining the real burden of disease. The Caribbean BOI studies will continue under CARPHA, with support from PAHO, using additional data being obtained in Antigua and Barbuda, Belize, and Bermuda.

I would like to thank the national authorities in each country for the support in conducting the studies; CAREC/CARPHA and PAHO secretariats for the coordination and management of country-specific studies and for support for publication of this JHPN Supplement. This work is made possible with the aid of a grant from the International Development Research Centre, Ottawa, Canada (www.idrc.ca) and the Cooperative Agreement of the Centers for Disease Control and Prevention, USA.

Again, thanks to all involved in this great work.

